# Synthesis of 3-*N*-Sugar-substituted-2, 4(1*H*, 3*H*)-quinazolinediones as Anti-Angiogenesis Agents

**DOI:** 10.3390/molecules14072447

**Published:** 2009-07-08

**Authors:** Conghai Huang, Xiangbao Meng, Jingrong Cui, Zhongjun Li

**Affiliations:** State Key Laboratory of Natural and Biomimetic Drugs, School of Pharmaceutical Sciences, Peking University, Beijing 100191, China; E-mails: michaelhchh@gmail.com (C.H.), xbmeng@bjmu.edu.cn (X.M.), jrcui@bjmu.edu.cn (J.C.)

**Keywords:** quinazolinediones, anti-angiogenesis, triphosgene

## Abstract

A series of novel 3-*N*-sugar-substituted quinazolinediones were synthesized through the cyclization of the intermediate 2-aminobenzamides using triphosgene as the condensing reagent. Their anti-angiogenesis activities were investigated. The compound 3-(2'-aminoglucosyl)-2,4-(1*H*,3*H*)-quinazolinedione, (**5d**) showed good anti-angiogenesis activity.

## 1. Introduction

The development of an effective anti-cancer drug is still a major challenge in the field of drug discovery. It was reported that aminopeptidase N (APN) plays a crucial role in the degradation and invasion of extracellular matrices by fibrosarcoma cells [1]_. _It is also important in the proliferation and the activation of pathogenic T-cells [2]. Several APN inhibitors were prepared to treat inflammatory disease, autoimmune disease, allogenic rejection reactions and allergies. In addition, APN antagonists were found to specifically inhibit angiogenesis in chorioallantoic membranes and in the retina, thus suppressing tumor growth. Therefore, APN was believed to be involved in angiogenesis and can serve as a target for the development of anti-cancer drugs [3,4,5].

**Figure 1:**
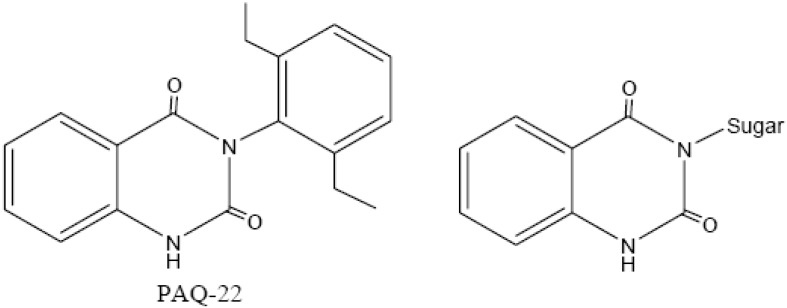
Structures of PAQ-22 and the desired 3-N-sugar substituted quinazolinedione derivatives.

The quinazolinediones have inhibitory activities towards some amino peptidases, such as puromycin-sensitive aminopeptidase (PSA) [5] and aminopeptidase N [1]. One representative compound, PAQ-22 (Figure 1), showed potent and specific PSA inhibiting activity with an IC_50_ of 0.09 μg/mL [5]. The inhibitory mechanism of these compounds was through non-competition, as revealed by the Lineweaver-Burk plot analysis. Structure-activity relationship studies indicated that tautomerism of the imidobenzoylketone group of the cyclic imide moiety of these kinds of inhibitors was important for the inhibitory activity [5].

It is reported that glucosamine, a type of amino-sugar, possesses immunosuppressive activity and could be beneficial as an immunosuppressive agent [6,7]. Water-soluble conjugates of glucosamine and glucosamine 6-sulfate were reported showing immunomodulatory and anti-angiogenesis properties, These derivatives of glucosamine could function synergistically to prevent scar tissue formation [8,9,10].

To find novel anti-angiogenesis agents, we have synthesized a series of 3-*N*-sugar-substituted-2,4-(1*H*,3*H*)-quinazolinediones containing amino-sugar moieties.

## 2. Results and Discussion

**Scheme 1:**
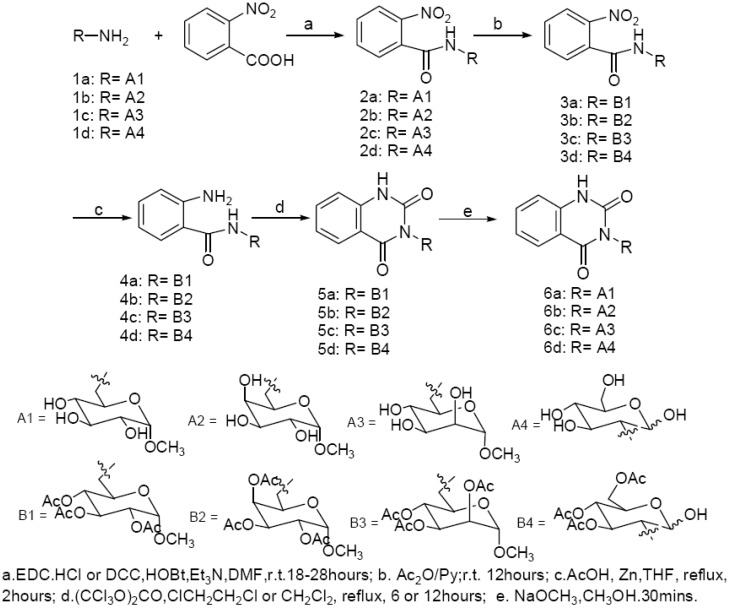
The synthetic route to *N*-sugar-substituted quinazolinedione derivatives.

Generally 3-*N*-substituted-2,4-(1*H*,3*H*)-quinazolinediones could be synthesized through different intermediates, including 2-amino benzamide [11], 2-ureayl benzoic ester [12,13,14], 2-isocyanato benzoate [15,16] and others [17,18]. Considering the known instability of quinazolinediones and the glucosamine moiety under acidic conditions, 2-amino-(*N*-sugar-substituted) benzamides were chosen as the key intermediates and a mild condensation condition was adopted in the synthetic route. During the synthesis of sugar-*N*^3^-substituted quinazolinediones, the unprotected aminosugar was used in the preparations of 2-amino-(*N*-sugar-substituted) benzamides. The aminosugars were prepared by the reported method [19]. The general synthesis of the key 2-nitro-(*N*-sugar-substituted) benzamide intermediates was readily performed by the condensation of 2-nitrobenzoic acid and unprotected aminosugars with EDC(DCC)/HOBt [20,21] (Scheme 1). After formation of amides, the sugar hydroxyl groups were fully acetyled by Ac_2_O/pyridine at room temperature, and then the nitro group was smoothly reduced by powdered Zn in acetic acid/THF. Finally, cyclization with triphosgene in CH_2_Cl_2_ (or ClCH_2_CH_2_Cl) was performed to produce the target molecules [20,21].

To prepare the intermediate 2-nitro-(*N*-sugar-substituted) benzamides from aminosugars, condensations of protected amino-sugars with 2-nitrobenzoyl chloride or with 2-nitrobenzoic acid activated by DCC (or EDC/HOBt) were attempted, but all these efforts failed. It was reported that the unprotected amino-sugars could be used directly to synthesize related amides [20,21], and following this method, the key *N*-sugar o-nitrobenzamide intermediates were obtained with isolated yields of 40-47%.

The *N*-sugar-substituted-2,4-(1*H*,3*H*)-quinazolinediones **5a-d** were obtained from the 2-amino-benzamides by carbonylation cyclization with carbonyldiimidazole (CDI), triphosgene and ethyl chlorocarbonate [20,21]. Several condensation conditions were tested. The results showed in Table 1 indicated that triphosgene had a higher activity than CDI and ethyl chlorocarbonate (Scheme 2). Therefore, triphosgene was used as the condensation reagent. After deacetylation with NaOMe/ MeOH, the target compounds **6a-d** were obtained in about 20% total yields. 

**Table 1:** Optimization of the conditions for the preparation of sugar-substituted quinazolinedine derivatives.

Entry	Reactant	Product	Reagent	Solvent	Reflux Time (h)	Isolated Yield (%)
*1*	4a	5a	CDI ^a^	THF	48	N.R ^b^
*2*	4a	5a	CDI ^a^	ClCH_2_CH_2_Cl	48	N.R ^b^
*3*	4a	5a	triphosgene	CH_2_Cl_2_	12	89%
*4*	4a	5a	triphosgene	ClCH_2_CH_2_Cl	6	85%
*5*	4b	5b	CDI ^a^	THF	48	N.R ^b^
*6*	4b	5b	ClCO_2_Et	CH_3_CN	48	N.R ^b^
*7*	4b	5b	triphosgene	CH_2_Cl_2_	12	84%
8	4b	5b	triphosgene	ClCH_2_CH_2_Cl	8	88%

^a ^CDI: carbonyldiimidazole; ^b ^N.R: no reaction.

**Figure 2:**
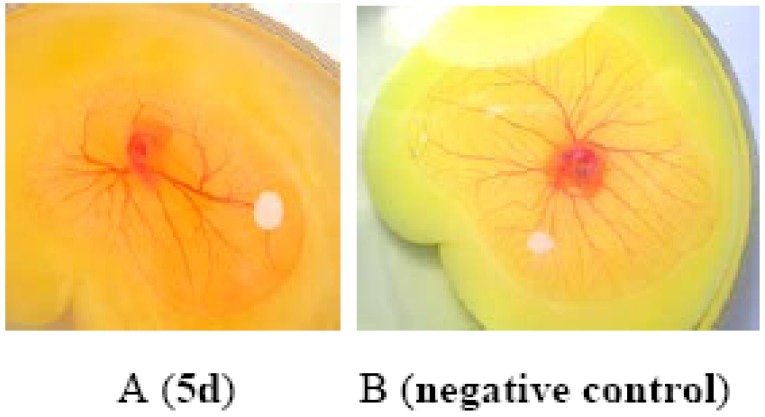
Inbibition of angiogenesis by compound **5d**.

The obtained *N*-sugar substituted-2,4-(1*H*,3*H*)-quinazolinediones **5a-d** and **6a-d** were primarily assayed for their angiogenesis inhibition activity using the chick chorioallantoic membrane (CAM) model [22]. Only compound **5d** showed good inhibitory activity to the neovascularization of chick *in vivo* (Figure 2).

## 3. Experimental

### 3.1. Instruments and apparatus

^1^H- and^ 13^C-NMR spectra were recorded on a Varian VXR 300 MHz spectrometer with Me_4_Si as the internal standard and CDCl_3 _or Me_2_SO-d_6 _as solvent. Optical rotations were measured at 25 °C with an AA-10R polarimeter. The progress of reactions was monitored by silica-gel GF_254_ TLC plates. Detection was performed by examination under UV light and by 15% H_2_SO_4 _in EtOH. Preparative TLC was performed on silica-gel GF_254_ plates and column chromatography was on silica-gel H. The inhibitory activity of anti-angiogenesic was assayed under chick chorioallantoic membrances (CAM) model, monitored by biological dissection microscope of DM-1 and recorded with a NIKON S610 digital camera.

### 3.2. Preparation of 3-N-sugar-substituted-2,4-(1H,3H)-quinazolinediones

#### 3.2.1. Synthesis and Spectral Data of **2a-c**

Aminosugar **1a-c** (2.9 g, 15 mmol), *o*-nitrobenzoic acid (3.2 g, 16.5 mmol) and 1-hydroxy benzotriazole (HOBt) (4.9 g, 36.3 mmol) were dissolved in DMF (80 mL). The mixture was cooled to 0 °C and stirred for 30 min. Then a solution of dicyclohexylcarbodiimide (DCC, 3.8 g, 18.2 mmol) in DMF (15 mL) was added dropwise. The mixture was stirred for 18 h at room temperature and filtered. The filtrate was evaporated to dryness under reduced pressure, and the residue was purified by column chromatography on silica gel to give **2a-c**.

*Methyl-6-(o-nitro)benzamidyl-6-deoxy-α-D-glucopyranoside* (**2a**). White flocculant crystals; Yield: 47.0%; mp: 230-233 °C; [a]_D_ +116^o^ (*c* 1.01, MeOH); ^1^H-NMR δ (ppm) (DMSO-d_6_): 3.18 (m, 1H, H-6b), 3.34 (m, 1H, H-2), 3.38 (s, 3H, OCH_3_), 3.47 (m, 2H, H-4,5), 3.61 (m, 1H, H-3), 3.85 (m, 1H, H-5), 4.51 (d, 1H, *J*_1, 2 _1.2Hz, H-1), 4.67 (d, 1H, *J* 6.0 Hz, H-OH), 4.79 (d, 1H, *J* 5.1 Hz, H-OH), 4.94 (d, 1H, *J* 5.4 Hz, H-OH), 7.55-8.03 (m, 4H, Ph), 8.76 (t, 1H, *J*5.1Hz, H-NH); ^13^C-NMR δ (ppm) (DMSO-d_6_): 40.8 (C-6), 54.4 (OCH_3_), 70.3 (C-5), 71.9 (C-3), 72.3 (C-2), 73.0 (C-4), 99.7 (C-1), 124.0 (Ph), 129.1 (Ph), 130.6 (Ph), 132.6 (Ph), 133.5 (Ph) , 147.1 (Ph), 165.1 (C=O); ESI-TOF-MS: [M+1]^+^ m/z 343.0; [M+Na]^+^ m/z 365.0.

*Methyl-6-(o-nitro)benzamidyl-6-deoxy-α-D-galactopyranoside* (**2b**). White flocculant crystals; Yield: 42.3%; mp: 218- 220 °C; [a]_D_ +52^o^ (*c* 1.01, DMSO); ^1^H-NMR δ (ppm) (DMSO-d_6_): 3.28 (s, 3H, OCH_3_), 3.01-3.77 (m, 5H, sugar-H), 4.57 (m, 3H, sugar-H), 7.57-8.03 (m, 4H, Ph), 8.82 (d, 1H, H-NH); ^13^C-NMR δ (ppm) (DMSO-d_6_): 45.5 (C-6), 54.6 (OCH_3_), 68.3 (C-2), 68.4 (C-5), 69.3 (C-4), 69.4 (C-3), 100.2 (C-1), 124.0 (Ph), 129.0 (Ph), 130.7 (Ph), 132.4 (Ph), 133.5 (Ph), 147.1 (Ph), 165.7 (C=O); ESI-TOF-MS: [M+1]^+^ m/z 343.0; [M+Na]^+^ m/z 365.0.

*Methyl-6-(o-nitro)benzamidyl-6-deoxy-α-D-mannopyranoside* (**2c**). White flocculant crystals; Yield: 47.0%; mp: 188-189 °C; [a]_D _+56^o^ (*c 1.01*, CHCl_3_);^ 1^H-NMR δ (ppm) (DMSO-d_6_): 3.13-3.49 (m, 5H, H-sugar), 3.39 (s, 3H, OCH_3_), 4.60 (m, 1H, H-sugar), 4.50 (s, 1H, H-1), 4.77 (d, *J* 8.7 Hz, H-OH), 4.78(d, *J* 4.8 Hz, H-OH), 4.92 (d, *J* 5.4 Hz, H-OH), 7.54-8.03 (m, 4H, Ph), 8.76 (t, 1H, *J* 5.1 Hz, H-NH); ^13^C-NMR δ (ppm) (DMSO-d_6_): 41.0 (C-6), 54.1 (OCH_3_), 69.6 (C-4), 70.2 (C-5), 70.6 (C-3), 71.4 (C-2), 101.0 (C-1), 124.0 (Ph), 129.1 (Ph), 130.6 (Ph), 132.6 (Ph), 133.5 (Ph), 147.1 (Ph), 165.9 (C=O); ESI-TOF-MS: [M+1]^+^ m/z 343.0; [M+Na]^+^ m/z 365.0.

#### 3.2.2. Synthesis and Spectral Data of 2-(*o*-nitro)benzamidyl-2-deoxy-β-D-glucopyranose (**2d**)

Glucosamine hydrochloride (7.8 g, 36 mmol) and sodium methoxide (2.25 g, 41.7 mmol) were added to methanol (100 mL). The mixture was stirred for 20 min and then evaporated to dryness under vacuum. The residue was dissolved in DMF (200 mL), followed by the addition of *o*-nitrobenzoic acid (5.1 g, 30 mmol) and 1-hydroxybenzotriazole (HOBt, 9.5 g, 72 mmol). The mixture was cooled to 0 °C and stirred for 30 min. Then the solution of dicyclohexylcarbodiimide (DCC, 6.9 g, 36 mmol) in DMF (25 mL) was added dropwise. The mixture was stirred for 20 h at room temperature and filtered. The filtrate was evaporated to dryness under reduced pressure, and the residue was purified through column chromatography on silica gel to yield 5.5 g of white flocculant crystals of **2d**; yield: 47%; mp: 208-212 °C; [a]_D_ +40^o^ (*c* 1.01, MeOH); ^1^H-NMR δ (ppm) (DMSO-d_6_): 3.28 (m, 1 H, H-sugar), 3.43-3.83 (m, 5 H, H-sugar), 4.55 (t, 1H, *J* 5.7 Hz, H-OH), 4.80 (d, 1H, *J*5.4Hz, H-OH), 5.05 (d, 1H, *J* 5.4 Hz, H-OH),5.18 (s, 1H, H-1), 6.62 (d, 1H, *J* 4.2 Hz H-OH), 7.73-8.10 (m, 4H, Ph), 8.64(d, 1H, *J* 8.1 Hz, H-NH); ^13^C-NMR δ(ppm) (75MHz, DMSO-d_6_): 55.1, 61.1, 70.1, 71.1, 72.1 (C of sugar ring), 90.4 (C-1), 123.8 (Ph), 129.5 (Ph), 130.6 (Ph), 132.4 (Ph), 133.1 (Ph), 147.3(Ph), 165.5(C=O); ESI-TOF-MS: [M+1]^+^ m/z 329.0; [M+Na]^+^ m/z 351.0.

#### 3.2.3. Synthesis and Spectral Data of **3a-d**

The appropriate 2-nitro-(*N*-sugar-substituted) benzamide **2a-d **(2.0 g) was dissolved in pyridine (50 mL), followed by the addition of acetic anhydride (25 mL). The solution was stirred at room temperature overnight and evaporated to dryness under reduced pressure. The residue was dissolved in ethyl acetate and washed sequentially with saturated sodium hydrogen carbonate solution, saturated brine and water. The organic layer was dried over anhydrous Na_2_SO_4_ and evaporated under reduced pressure to give **3a-d **as yellow solids.

*Methyl-6-(o-nitro-)benzamidyl-6-deoxy-2,3,4-tri-O-acetyl-α-D-glucopyranoside* (**3a**). Yield: 95.0 %; mp: 77-79 ^o^C; [a]_D _+104 ^o^ (*c* 1.01, CHCl_3_); ^1^H-NMR δ (ppm) (CDCl_3_): 2.02 (s, 3H, Ac), 2.08 (s, 3H, Ac), 2.10 (s, 3H, Ac), 3.42 (s, 3H, OCH_3_), 3.61 (m, 1H, H-6), 3.79 (m, 1H, H-5), 4.04 (m, 1H, H-5), 4.83 (dd, 1H, *J*_1,2 _3.6 Hz, *J*_2,3_ 10.2 Hz, H-2), 4.91 (d, 1H, *J*_1, 2 _3.6 Hz, H-1), 5.02 (t, 1H, *J* 9.9 Hz, H-3), 5.48 (t, 1H, *J* 9.9 Hz, H-4), 6.26 (t, 1H, *J* 5.7 Hz, H-NH), 7.27-8.09 (m, 4H, Ph); ^13^C-NMR δ (ppm) (CDCl_3_): 20.7 (CH_3_CO), 39.4 (C-6), 55.0 (OCH_3_), 67.6 (C-5), 69.2 (C-3), 69.8 (C-2), 70.9 (C-4), 96.8 (C-1), 124.5 (Ph), 129.0 (Ph), 130.5 (Ph), 132.7 (Ph), 133.9 (Ph), 146.2 (Ph), 166.6 (C=O), 169.9 (CH_3_CO), 170.2 (CH_3_CO), 170.3 (CH_3_CO); ESI-TOF-MS: [M+1]^+^ m/z 469.1; [M+Na]^+^ m/z 491.1.

*Methyl-6-(o-nitro-)benzamidyl-6-deoxy-2,3,4-tri-O-acetyl-α-D-galactopyranoside* (**3b**). Yield: 98.0%; mp: 87-90 °C; [a]_D_+68^o^ (*c 1.01*, CHCl_3_); ^1^H-NMR δ (ppm) (CDCl_3_): 1.99 (s, 3H, Ac), 2.07 (s, 3H, Ac), 2.18 (s, 3H, Ac), 3.42(s, 3H, OCH_3_), 3.54 (m, 2H, H-6), 4.22 (t, 1H, *J* 6.6 Hz, H-5), 4.98 (d, 1H, *J*_1, 2 _3.0 Hz, H-1), 5.16 (dd, 1H, *J*_1,2 _3.0 Hz, *J*_2,3 _10.8 Hz, H-2), 5.37-5.46 (m, 2H, H-4, H-3), 6.42 (br, 1H, H-NH), 7.38-8.06 (m, 4H, Ph); ^13^C-NMR δ (ppm) (CDCl_3_): 20.6 (CH_3_CO), 20.7 (CH_3_CO), 20.8 (CH_3_CO), 39.3 (C-6), 55.7 (OCH_3_), 66.5 (C-5), 67.4 (C-3), 68.2 (C-2), 69.0 (C-4), 97.2 (C-1), 124.5 (Ph), 128.6 (Ph), 130.6 (Ph), 132.5 (Ph), 133.8 (Ph), 146.4 (Ph), 166.6 (C=O), 169.7 (CH_3_CO), 170.4 (CH_3_CO), 170.8 (CH_3_CO); ESI-TOF-MS: [M+Na]^+^ m/z 491.0.

*Methyl-6-(o-nitro-)benzamidyl-6-deoxy-2,3,4-tri-O-acetyl-α-D-mannopyranoside* (**3c**). Yield: 91.3%; mp:162-164 °C; [a]_D_+28^o^ (*c* 1.10, CHCl_3_); ^1^H-NMR δ (ppm) (CDCl_3_): 2.00 (s, 3H, Ac), 2.11 (s, 3H, Ac), 2.14 (s, 3H, Ac), 3.38 (s, 3H, OCH_3_), 3.60 (m, 1H, H-6e), 3.78 (m, 2H, H-6a, 5), 4.67 (s, 1H, H-1), 5.20-5.34 (m, 3H, H-2, 3, 4), 6.40 (t, 1H, *J* 6.6 Hz, H-NH), 7.58-8.09 (m, 4H, Ph); ^13^C-NMR δ (ppm) (CDCl_3_): 20.7(CH_3_CO), 20.8 (CH_3_CO), 39.8 (C-6), 55.3 (OCH_3_), 66.8 (C-4), 68.8 (C-5), 68.9 (C-3), 69.6 (C-2), 98.5 (C-1), 124.6 (Ph), 128.7 (Ph), 130.6 (Ph), 132.9 (Ph), 133.7 (Ph), 146.3(Ph), 166.4(C=O), 169.8 (CH_3_CO), 169.9 (CH_3_CO), 170.4 (CH_3_CO); ESI-TOF-MS: [M+1]^+^ m/z 469.0; [M+Na]^+^ m/z 491.0.

*2-(o-Nitro)benzamidyl-2-deoxy-1,3,4,6-tetra-O-acetyl-β-D-glucopyranose* (**3d**). Yield: 93.0%; mp: 144-148 °C; [a]_D _+40^o^ (*c 1.01*, CHCl_3_);^ 1^H-NMR δ (ppm) (CDCl_3_): 2.06 (s, 3H, Ac), 2.11 (s, 3H, Ac), 2.14 (s, 3H, Ac), 2.17 (s, 3H, Ac), 4.05-4.13 (m, 2H, H-6), 4.30 (dd, 1H, *J* 3.6 Hz, 12.6 Hz, H-4), 4.67 (m, 1H, H-5), 5.30 (m, 2H, H-2,3), 6.14 (d, 1H, *J* 8.4 Hz, H-1), 6.40 (d, 1H, *J* 3.6 Hz, H-NH), 7.27-8.09 (m, 4H, Ph);^13^C-NMR δ (ppm) (CDCl_3_): 20.5 (CH_3_CO), 20.7 (CH_3_CO), 20.8 (CH_3_CO), 20.9 (CH_3_CO), 51.9 (OCH_3_), 61.4 (C-sugar), 67.4 (C-sugar), 69.8 (C-sugar), 70.0 (C-sugar), 90.4(C-1), 124.6 (Ph), 128.7 (Ph), 130.8 (Ph), 132.0 (Ph), 134.0 (Ph), 145.9 (Ph), 166.5 (C=O), 168.7 (CH_3_CO), 169.9 (CH_3_CO), 170.7 (CH_3_CO), 172.4 (CH_3_CO); ESI-TOF-MS: [M+Na]^+^ m/z 519.1.

#### 3.2.4. Synthesis and Spectral Data of **4a-c**

The appropriate compound **3a-c** (2.0 g, 7.4 mmol) was dissolved in THF (50 mL) and acetic acid (5 mL). Under stirring, zinc power (1.3 g, 20 mmol) was added slowly. The mixture was then refluxed for 2 h, cooled to room temperature, and filtered through a short column of silica gel. The eluent was evaporated to dryness under vacuum. The residue was dissolved in ethyl acetate and washed sequentially with saturated sodium hydrogen carbonate solution, saturated brine and water. The organic layer was dried over anhydrous Na_2_SO_4_ and evaporated under reduced pressure to afford the compounds **4a-c **as yellow solids.

*Methyl-6-(o-amino)benzamidyl-6-deoxy-2,3,4-tri-O-acetyl-α-D-glucopyranoside* (**4a**). Yield: 88.0%; mp: 108-110 °C; [a]_D _+98^o^ (*c 1.01*, CHCl_3_); ^1^H-NMR δ (ppm) (CDCl_3_): 2.01 (s, 3H, Ac), 2.07 (s, 3H, Ac), 2.09 (s, 3H, Ac), 3.43 (s, 3H, OCH_3_), 3.61 (m, 1H, H-6), 3.79 (m, 1H, H-5), 4.04 (m, 1H, H-6), 4.83 (dd, 1H, *J*_1,2 _3.6 Hz, *J*_2,3_ 9.6 Hz, H-2), 4.93 (m, 2H, H-1, 3), 5.50 (t, 1H, *J* 9.6 Hz, H-3), 5.48 (t, 1H, *J* 9.9 Hz, H-4), 6.45 (t, 1H, H-NH), 6.45-7.36 (m, 4H, Ph); ^13^C-NMR δ (ppm) (CDCl_3_): 20.7 (CH_3_CO), 22.6 (CH_3_CO), 38.9 (C-6), 55.4 (OCH_3_), 67.7 (C-5), 69.7 (C-3), 69.9 (C-2), 70.9 (C-4), 96.6 (C-1), 112.2 (Ph), 114.1 (Ph), 127.4 (Ph), 132.9 (Ph), 148.8 (Ph), 169.7 (C=O), 170.0 (CH_3_CO), 170.1 (CH_3_CO), 170.2 (CH_3_CO); ESI-TOF-MS: [M+1]^+^ m/z 439.1; [M+Na]^+^ m/z 461.1.

*Methyl-6-(o-amino)benzamidyl-6-deoxy-2,3,4-tri-O-acetyl-α-D-galactopyranoside* (**4b**). Yield: 78.0%; mp: 78-80 °C; [a]_D _+28^o^ (*c 1.01*, CHCl_3_); ^1^H-NMR δ (ppm) (CDCl_3_): 2.00 (s, 3H, Ac), 2.10 (s, 3H, Ac), 2.20 (s, 3H, Ac), 3.39 (s, 3H, OCH_3_), 3.44 (t, 1H, *J* 6.6 Hz, H-5), 3.60 (dd, 1H, *J*_5,6e_ 6.9 Hz, *J*_6a__,6e_ 13.5 Hz, H-6e), 4.15 (dd, 1H, *J*_5__,__6a_ 6.9 Hz, *J*_6a__, 6e _13.5 Hz, H-6a), 5.00 (d, 1H, *J*_1, 2 _3.6 Hz, H-1), 5.18 (dd, 1H, *J*_1,2 _3.6 Hz, *J*_2,3 _10.8 Hz, H-2), 5.37 (dd, 1H, *J*_3,4 _3.3 Hz, *J*_2,3 _10.8 Hz, H-3), 5.45 (d, 1H, *J* 3.3 Hz, H-4), 6.49 (t, 1H, *J* 6.3 Hz, H-NH), 6.63-7.33 (m, 4H, Ph); ^13^C-NMR δ (ppm) (CDCl_3_): 20.6 (CH_3_CO),20.7 (CH_3_CO), 20.8 (CH_3_CO), 38.8 (C-6), 55.0 (OCH_3_), 66.7 (C-5), 67.5 (C-3), 68.3 (C-2), 69.4 (C-4), 97.2 (C-1), 115.2 (Ph), 116.7 (Ph), 117.4 (Ph), 127.0 (Ph), 132.5 (Ph), 148.9 (Ph), 169.2 (C=O), 169.8 (CH_3_CO) , 170.5 (CH_3_CO), 171.0 (CH_3_CO); ESI-TOF-MS: [M+1]^+^ m/z 439.1; [M+Na]^+^ m/z 461.1.

*Methyl-6-(o-amino)benzamidyl-6-deoxy-2,3,4-tri-O-acetyl-α-D-mannopyranoside* (**4c**). Yield: 77.6%; mp: 150-154 °C; [a]_D _+40^o^ (*c* 1.10, CHCl_3_); ^1^H-NMR δ (ppm) (CDCl_3_): 2.00 (s, 3H, Ac), 2.10 (s, 3H, Ac), 2.12 (s, 3H, Ac), 3.37 (s, 3H, OCH_3_), 3.40 (m, 1H, H-6e), 3.85-3.98 (m, 2H, H-5, 6a), 4.70 (s, 1H, H-1), 5.19-5.25 (m, 2H, H-2, 4), 5.36 (dd, 1H, *J*_3,2 _3.3 Hz, *J*_3,4 _10.8 Hz, H-3), 6.56 (t, 1H, *J* 5.4 Hz, H-NH), 6.63-7.37 (m, 4H, Ph); ^13^C-NMR δ (ppm) (CDCl_3_): 20.7 (CH_3_CO), 20.8 (CH_3_CO), 39.3 (C-6), 55.3 (OCH_3_), 67.1 (C-4), 68.8 (C-5), 68.9 (C-3), 69.6 (C-2), 98.4 (C-1), 115.8 (Ph), 116.5 (Ph), 117.3 (Ph), 126.9 (Ph), 132.4 (Ph), 148.8 (Ph), 169.2(C=O), 169.9 (CH_3_CO), 170.0 (CH_3_CO), 170.3 (CH_3_CO); ESI-TOF-MS: [M+1]^+^ m/z 439.1; [M+Na]^+^ m/z 461.1.

#### 3.2.5. Synthesis and Spectral Data of **4d**

Compound **3d** (200 mg, 0.4 mmol) was dissolved in methanol (30 mL), and 40% Pd(OH)_2 _(20 mg) was added. Catalytic hydrogenation was carried out at 4.5 atm of pressure for 6 hours. The solid was filtered and the filtrate was evaporated to dryness to afford 180 mg of **4d**, yield: 95.0%; mp: 158-160 °C;^ 1^H-NMR δ (ppm) (CDCl_3_): 2.05 (s, 3H, Ac), 2.07 (s, 3H, Ac), 2.11 (s, 3H, Ac), 2.18 (s, 3H, Ac), 4.01-4.16 (m, 2H, H-5, 6), 4.30 (dd, 1H, *J* 3.6 Hz, 12.3Hz ), 4.67 (m, 1H, H-5), 5.26-5.42 (m, 2H), 6.25 (d, 1H, *J* 8.7 Hz, H-1), 6.31 (d, 1H, *J* 3.6 Hz, H-NH), 7.27-8.09 (m, 4H, Ph); ^13^C-NMR δ (ppm) (CDCl_3_) 20.6 (CH_3_CO), 20.7 (CH_3_CO), 20.8 (CH_3_CO), 20.9 (CH_3_CO), 51.3 (OCH_3_), 61.5, 67.4, 69.7, 70.6(C of sugar ring), 90.6 (C-1), 114.3 (Ph), 116.7 (Ph), 117.4 (Ph), 127.0 (Ph), 132.9 (Ph), 149.0 (Ph), 168.7 (C=O), 168.8 (CH_3_CO), 169.1 (CH_3_CO), 170.7 (CH_3_CO), 172.1 (CH_3_CO); ESI-TOF-MS: [M+1]^+^ m/z 467.1; [M+Na]^+^ m/z 489.1.

#### 3.2.6. Synthesis and Spectral Data of **5a-d**

Compounds **4a-d **(300 mg) were dissolved in ClCH_2_CH_2_Cl (50 Ll), then triphosgene (140 mg, 0.54 mmol) was added. The mixture was refluxed for 6h and cooled to room temperature. CH_2_Cl_2_ (50 mL) was added and the organic layer was washed with saturated sodium hydrogen carbonate solution, saturated brine and water. The organic layer was dried over anhydrous Na_2_SO_4,_ evaporated under reduced pressure to dryness, and purified with column chromatography on silica gel to yield white solids of **5a-d**.

*Methyl-6-(N^3^-)quinazolinedionyl-6-deoxy-2,3,4-tri-O-acetyl-α-D-glucopyranoside* (**5a**). Yield: 89.0%; mp: 118-120 °C; [a]_D _+108^o^ (*c 1.01*, CHCl_3_); ^1^H-NMR δ (ppm) (CDCl_3_): 2.01 (s, 3H, Ac), 2.05 (s, 3H, Ac), 2.18 (s, 3H, Ac), 3.20 (s, 3H, OCH_3_), 4.14 (dd, 1H, *J*_6e, 5 _3.9Hz, *J*_6a__, 6e _13.5 Hz, H-6e), 4.30 (m, 1H, H-5), 4.51 (dd, 1H, *J*_6a__, 5 _8.4 Hz, *J*_6a__, 6e _12.9 Hz, H-6a), 4.90 (d, 1H, *J*_1, 2 _3.6 Hz, H-1), 4.94 (dd, 1H, *J*_1,2_ 8.4 Hz, *J*_2,3_12.9 Hz, H-2),5.09 (t, 1H, *J* 9.3 Hz, H-4), 5.48 (t, 1H, *J* 9.3 Hz, H-3), 7.16-8.16 (m, 4H, Ph). 10.2 (s, 1H, H-NH);^ 13C^-NMR δ (ppm) (CDCl_3_): 20.7 (CH_3_CO), 39.4 (C-6), 55.0 (OCH_3_), 67.6 (C-5), 69.2 (C-3), 69.8 (C-2), 70.9 (C-4), 96.8 (C-1), 124.5 (Ph), 129.0 (Ph), 30.5 (Ph), 132.7 (Ph), 133.9 (Ph), 146.2 (Ph), 166.6 (C=O), 169.9 (CH_3_CO), 170.2 (CH_3_CO), 170.3 (CH_3_CO); ESI-TOF-MS: [M+1]^+^ m/z 465.0; [M+Na]^+^ m/z 487.0.

*Methyl-6-(N^3^-)quinazolinedionyl-6-deoxy-2,3,4-tri-O-acetyl-α-D-galactopyranoside* (**5b**). Yield: 89.0%, mp: 216-219 °C; [a]_D _+220^o^ (*c 1.01*, CHCl_3_); ^1^H-NMR δ (ppm) (CDCl_3_): 1.96 (s, 3H, Ac), 2.08 (s, 3H, Ac), 2.26 (s, 3H, Ac), 3.34 (s, 3H, OCH_3_), 4.29 (m, 2H, H-5,6e), 4.52 (t, 1H, *J* 6.6 Hz, H-6a), 5.00 (d, 1H, *J*_1, 2 _3.3 Hz, H-1), 5.20 (dd, 1H, *J*_1,2 _3.3 Hz, *J*_2,3 _10.8 Hz, H-2), 5.33 (dd, 1H, *J*_3,4 _3.3 Hz, *J*_2,3 _10.8 Hz, H-3), 5.38 (d, 1H, *J* 3.0 Hz, H-4), 7.10-8.13 (m, 4H, Ph), 10.1 (s, 1H, H-NH); ^13^C-NMR δ (ppm) (CDCl_3_): 20.7 (s, 3H, Ac), 20.8 (s, 3H, Ac), 20.9 (CH_3_CO), 39.8 (C-6), 55.2 (OCH_3_), 65.2 (C-5), 67.9 (C-3), 68.1 (C-2), 68.2 (C-4), 97.0 (C-1), 114.2 (Ph), 115.0 (Ph), 123.6 (Ph), 128.5 (Ph), 135.3 (Ph), 138.4 (Ph), 151.6 (C=O), 162.2 (C=O), 170.1 (CH_3_CO), 170.4 (CH_3_CO), 170.8 (CH_3_CO); ESI-TOF-MS: [M+1]^+^ m/z 465.1; [M+Na]^+^ m/z 487.1.

*Methyl-6-(N^3^-)quinazolinedionyl-6-deoxy-2,3,4-tri-O-acetyl-α-D-mannopyranoside* (**5c**). Yield: 81.8%; mp: 78-82 °C; [a]_D _+32^o^ (*c 1.01*, CHCl_3_);^ 1^H-NMR δ (ppm) (CDCl_3_): 2.01 (s, 3H, Ac), 2.07, (s, 3H, Ac) 2.16 (s, 3H, Ac), 3.19 (s, 3H, OCH_3_), 4.14 (dd, 1H, *J*_6e, 5 _3.9 Hz, *J*_6a__, 6e _13.5 Hz, H-6e), 4.30 (m, 1H, H-5), 4.51 (dd, 1H, *J*_6a__, 5 _8.4 Hz, *J*_6a__, 6e _13.5 Hz, H-6a), 4.60 (dd, 1H, *J*_2,3 _2.1 Hz, *J*_1,2 _4.8 Hz, H-2), 5.21 (d, 1H, *J*_1, 2 _2.1 Hz, H-1), 5.32-8.34 (m, 2H, H-4,3), 7.21-8.16 (m, 4H, Ph). 10.7 (s, 1H, H-NH); ^13^C-NMR δ (ppm) (CDCl_3_): 20.6 (CH_3_CO), 20.7 (CH_3_CO), 20.9 (CH_3_CO), 41.9 (C-6), 54.8 (OCH_3_), 67.3 (C-5), 69.0 (C-3), 69.1 (C-2), 69.5 (C-4), 98.1 (C-1), 114.1 (Ph), 115.2 (Ph), 123.5 (Ph), 128.4 (Ph), 135.2 (Ph), 138.5 (Ph), 152.0 (C=O), 162.2 (C=O), 169.9 (CH_3_CO), 170.1 (CH_3_CO), 170.2 (CH_3_CO); ESI-TOF-MS: [M+1]^+^ m/z 465.0; [M+Na]^+^ m/z 487.0.

*2-(N^3^-)quinazolinedionyl-2-deoxy-1,3,4,6-tetra-O-acetyl-β-D-glucopyranoside* (**5d**). Yield: 57.1%; mp: 193-195 °C; [a]_D _+88^o^ (*c 1.01*, CHCl_3_);^ 1^H-NMR δ (ppm) (CDCl_3_): 2.04 (s, 3H, Ac), 2.05 (s, 3H, Ac), 2.11 (s, 3H, Ac), 2.18 (s, 3H, Ac), 4.02-4.32 (m, 4H, H-sugar), 4.67 (m, 1H, H-5), 5.26-5.42 (m, 2H, H-sugar), 6.33 (d, 1H, *J* 3.6 Hz, H-sugar), 6.37 (d, 1H, *J* 8.1 Hz, H-1), 7.01-8.39 (m, 4H, Ph), 10.2 (ds, 1H, H-NH); ^13^C-NMR δ (ppm) (CDCl_3_): 20.6 (CH_3_CO), 20.7 (CH_3_CO), 20.8 (CH_3_CO), 20.9 (CH_3_CO), 51.8 (OCH_3_), 61.2, 61.5, 67.2, 69.7, 70.6 (C of sugar ring), 90.4 (C-1), 118.3 (Ph), 121.9 (Ph), 126.3 (Ph), 133.3 (Ph), 140.4 (Ph), 153.8 (Ph), 168.6 (C=O), 169.1 (CH_3_CO), 172.1 (CH_3_CO), 179.9 (C=O); ESI-TOF-MS: [M+NH_4_]^+^ m/z 510.0; [M+Na]^+^ m/z 515.0; [M+K]^+^ m/z 530.9.

#### 3.2.7. Synthesis and Spectral Data of **6a-d**

The appropriate intermediate **5a-d **(130 mg) was dissolved in MeOH(20 mL) and sodium methoxide (10 mg, 0.18 mmol) was added and the mixture stirred for 30 min. The solution was then neutralized to pH 6-7 by with resin and filtered. The filtrate was evaporated to dryness to obtain light yellow solid of **6a-d.**

*Methyl-6-(N^3^-)quinazolinedionyl-6-deoxy-α-D-glucopyranoside* (**6a**). Yield: 98%; mp: 137-142 °C; [a]_D_ +60^o^ (*c* 1.01, DMSO); ^1^H-NMR δ (ppm) (DMSO-d_6_): 2.97 (s, 3H, OCH_3_), 3.03 (m, 1H), 3.17 (m, 1H), 3.35 (m, 1H), 3.82 (m, 1H), 4.05 (m, 1H), 4.19 (m, 1H), 4.40 (d, 1H, *J*_1, 2_ 3.0 Hz, H-1); ^13^C-NMR δ (ppm) (DMSO-d_6_): 41.8 (C-6), 53.7 (OCH_3_), 67.2 (C-5), 71.9 (C-3), 73.2 (C-2), 73.8 (C-4), 99.5 (C-1), 113.8 (Ph), 115.8 (Ph), 122.0 (Ph), 27.3 (Ph), 134.7 (Ph), 140.7(Ph), 150.9 (C=O), 162.3 (C=O); ESI-TOF-MS: [M+1]^+^ m/z 339.0; [M+Na]^+^ m/z 361.0.

*Methyl-6-(N^3^-)quinazolinedionyl-6-deoxy-α-D-galactopyranoside* (**6b**). Yield: 81.5%; mp: 235-237 °C; [a]_D _+220^o^ (*c 1.01*, MeOH);^ 1^H-NMR δ (ppm) (DMSO-d_6_): 3.34 (s, 3H, OCH_3_), 3.49-3.58 (m, 2H), 3.65-3.96 (m, 3H), 4.07 (dd, 1H, 1H, *J*_2,3 _3.6 Hz, *J*_3,4 _9.3 Hz, H-3), , 4.37 (s, 1H, H-1), 4.42-4.59 (m, 3H); ^13^C-NMR δ (ppm) (DMSO-d_6_): 40.9 (C-6), 55.2 (OCH_3_), 67.1, 68.2, 69.0, 70.2 (C of sugar ring), 99.9 (C-1), 104.2 (C-1), 113.7 (Ph), 115.1 (Ph), 122.4 (Ph), 127.4 (Ph), 135.0 (Ph), 139.4(Ph), 150.4 (C=O), 162.2 (C=O); ESI-TOF-MS: [M+1]^+^ m/z 339.1; [M+Na]^+^ m/z 361.0.

*Methyl-6-(N^3^-)quinazolinedionyl-6-deoxy-α-D-mannopyranoside* (**6c**). Yield: 81.0%; mp: 169-172^o^C; [a]_D_ +60^o^ (*c* 1.01, DMSO); ^1^H-NMR δ (ppm) (DMSO-d_6_): 2.93 (s, 3H, OCH_3_), 3.39-3.54 (m, 3H, H-5, 6a, 6e), 3.76 (m, 1H), 4.06 (dd, 1H, 1H, *J*_2,3 _3.6 Hz, *J*_3,4 _9.3 Hz, H-3), 4.27 (dd, 1H, *J*_4,5 _9.6 Hz, *J*_3,4 _13.2 Hz, H-4), 4.37 (s, 1H, H-1); ^13^C-NMR δ (ppm) (DMSO-d_6_): 41.8 (C-6), 53.4 (OCH_3_), 68.2, 70.1, 70.9 (C of sugar ring), 100.8 (C-1), 113.7 (Ph), 115.1 (Ph), 122.4 (Ph), 127.4 (Ph), 134.9 (Ph), 139.5 (Ph), 150.3 (C=O), 162.1 (C=O); ESI-TOF-MS: [M+1]^+^ m/z 339.0; [M+Na]^+^ m/z 361.0.

*2-(N^3^-)quinazolinedionyl-2-deoxy-D-glucopyranoside* (**6d**). Yield: 90.9%; mp:174-179 °C; [a]_D _+20^o^ (*c* 1.10, DMSO); ^1^H-NMR δ (ppm) (DMSO-d_6_): 3.69-3.78 (m, 2H, H-5, H-6), 4.01 (dd, 1H, *J*_6a__, 5 _3.0 Hz, *J*_6a__, 6e _5.4 Hz, H-6a), 4.60 (t, 1H, *J* 4.8 Hz), 4.78 (dd, 1H, *J*_2,3 _2.4 Hz, *J*_2,1 _5.1 Hz, H-2), 5.08 (dd, 1H, *J*_2,3 _2.4 Hz, *J*_3,41 _5.1 Hz, H-3), 5.54 (d, 1H, *J*_2,1 _5.1 Hz, H-1), 6.0 (s, 1H, H-OH), 7.18-7.95 (m, 4H, Ph); ^13^C-NMR δ (ppm) (DMSO-d_6_): 65.9, 69.3, 70.9, 80.6, 84.6 (C-sugar), 100.8 (C-1), 113.7 (Ph), 115.1 (Ph), 122.7 (Ph), 127.5 (Ph), 135.2 (Ph), 139.5 (Ph), 150.0 (C=O), 162.1 (C=O); ESI-TOF-MS: [M+NH_4_]^+^ m/z 338.0; [M+Na]^+^ m/z 347.0.

### 3.3. Anti-angiogenesic Inhibitory Activity of the Target Compounds

The eggs were cut and chicken embryos were incubated under 37.5 °C for 7 days. When the CAM‘s diameter had grown to 1-3cm, solutions of the compounds was added to each chicken embryo with PBS as control. The results were recorded by camera under a dissection microscope [22].

## 4. Conclusions

In summary, several novel 3-*N*-sugar-substituted quinazolinediones were synthesized and their anti-angiogenesis activities were tested. An efficient method, using triphosgene as the carbonylation condensation reagent, was developed for the synthesis of *N*-sugar-substituted quinazolinediones. This method might be useful in the future for the preparation of similar derivatives.
